# High Proportion of Asymptomatic SARS-CoV-2 Infections in 9 Long-Term Care Facilities, Pasadena, California, USA, April 2020

**DOI:** 10.3201/eid2610.202694

**Published:** 2020-10

**Authors:** Matt Feaster, Ying-Ying Goh

**Affiliations:** City of Pasadena Public Health Department, Pasadena, CA, USA

**Keywords:** COVID-19, skilled nursing facility, assisted living facility, long-term care facilities, 2019 novel coronavirus disease, severe acute respiratory syndrome coronavirus 2, zoonoses, coronavirus disease, SARS-CoV-2, California, United States

## Abstract

Our analysis of coronavirus disease prevalence in 9 long-term care facilities demonstrated a high proportion (40.7%) of asymptomatic infections among residents and staff members. Infection control measures in congregate settings should include mass testing–based strategies in concert with symptom screening for greater effectiveness in preventing the spread of severe acute respiratory syndrome coronavirus 2.

Severe acute respiratory syndrome coronavirus 2 (SARS-CoV-2) is a novel human coronavirus that causes coronavirus disease (COVID-19). The disease was detected in the United States on January 20, 2020, and had caused >1.7 million cases and >100,000 deaths as of June 1, 2020 (*1*,*2*). As the pandemic continues, data consistently show that older adults, particularly those with >1 underlying medical conditions, experience higher hospitalization rates and increased vulnerability to in-hospital death (*3*,*4*).

Long-term care facilities (LTCFs) in the United States, including skilled nursing facilities (SNFs) and assisted living facilities (ALFs), are populated by older adults and adults needing residential care for underlying medical conditions who are at increased risk of more severe COVID-19–associated illness (*4*,*5*). ALF residents generally require a limited amount of care, such as help getting dressed or assistance with medications, whereas SNF residents have acute or chronic health conditions, or both, that require 24-hour onsite medical care and often rehabilitative care and therapy. 

The city of Pasadena, California, USA, is an independent local public health jurisdiction that has a disproportionately high representation of older adults compared with other southern California local public health jurisdictions. More than 12.0% of Pasadena’s population is >70 years of age, compared with 9.1% in Los Angeles County (*6*), and Pasadena has >1,253 licensed SNF beds, which is 2.4 times the rate (per 100,000 residents) of SNF beds as in the Los Angeles County public health jurisdiction (*7*). The Pasadena Public Health Department (PPHD) recognized that this large population of medically fragile adults was at high risk for illness and death from COVID-19 as it spread through California, especially after early reports of nursing facility outbreaks in late February (*8*).

Extensive COVID-19–specific outreach and education efforts with skilled nursing facilities by PPHD staff began in late January 2020. In the second week of March, the PPHD received a report of laboratory-confirmed COVID-19 in a Pasadena resident (not facility-associated); a report of COVID-19 in a LTCF employee was received on March 31. By mid-April, the PPHD had opened investigations for facilities with >1 confirmed COVID-19 case in 14 of 15 SNFs in the city jurisdiction and 3 of 26 ALFs. By the end of April, 19 facilities in Pasadena had completed mandatory facilitywide screenings for SARS-CoV-2 to aid in the investigation and control of COVID-19 transmission.

## The Study

Facilitywide testing of staff and residents was completed in all facilities by the end of April, with thousands of test results available by early May. This analysis was restricted to facilities with >3 linked cases. Facilities excluded from the analysis had singular cases, non–epidemiology-linked cases, or no reported COVID-19 cases at the time of the initial mass testing. Of the 19 facilities, 9 (8 SNFs and 1 ALF) had evidence of sustained transmission by investigation within the facility and were included in this analysis. Residents were included if they were listed on the facility’s census sheet on the day the investigation was opened. All types of staff, both clinical and nonclinical, were required to participate.

A case-patient was defined as a person with a nasopharyngeal swab specimen that tested positive for SARS-CoV-2 by real-time reverse transcription PCR (rRT-PCR) (*9*) at a commercial laboratory or the Los Angeles County Public Health Laboratory (Downey, CA, USA). Laboratory results were combined with case investigation data collected by PPHD public health nurses. Symptom data were extracted from case reports compiled during the case investigation (*10*), patient medical records (hospital and physician notes), and facility clinical staff assessments and records for residents. Residents and staff were classified as symptomatic if they had had >1 new or worsened signs or symptoms of COVID-19 in the 14 days before nasal swab specimen collection. Persons with subjective fever or temperature >100.0°F (37.8°C), muscle aches, cough, shortness of breath, fatigue, headache, new loss of taste or smell, sore throat, runny nose, nausea or vomiting, diarrhea, low oxygen saturation, or clinical oxygen requirement (as determined by the patient’s physician) were classified as symptomatic (*11*).

A total of 1,093 persons (608 residents and 485 staff members) were eligible for rRT-PCR testing for SARS-CoV-2 based on facilitywide testing strategies at the 9 LTCF sites (Table 1). Test results for 85.9% (938/1,093) of the staff and residents were obtained by PPHD, specifically 95.7% (582/608) of residents and 73.6% (356/485) of staff. The overall population (residents and staff) prevalence of SARS-CoV-2 among these 9 facilities was 67.3% (631/938). The overall prevalence of asymptomatic infection among those who tested positive was 40.7% (257/631). The prevalence of SARS-CoV-2 infection among staff involved with direct patient care, such as certified nursing assistants (CNAs), licensed vocational nurses (LVNs), registered nurses (RNs), and other caregivers (68.5%, 150/219) was higher than among those not providing direct patient care, such as activity, dietary, and maintenance workers (48.1%, 25/52). A larger percentage of female staff (62.5%) than male staff (46.5%) functioned in clinical roles. The prevalence of SARS-CoV-2 infection among all residents was 70.1% (408/582); among female residents, the prevalence was 71.4% (237/332), and among male residents, it was 68.4% (171/250). Female residents had a higher rate of asymptomatic infection (51.0%, 121/237) than male residents (47.4%, 81/171).

**Table 1:** Demographics and COVID-19 characteristics by staff and residents among long-term care facilities, Pasadena, California, USA, April 2020*

Characteristic	Total eligible	Persons tested	Confirmed COVID-19†	Asymptomatic infection†
Staff	485	356 (73.4)	223 (62.6)	55 (24.7)
Age, y, mean (SD)	443	41.8 (13.3)	42.8 (12.7)	39.8 (14.2)
Sex				
F	328	249 (75.9)	170 (68.3)	39 (22.9)
M	157	107 (68.2)	53 (49.5)	16 (30.2)
Staff role				
Activities	15	9 (60.0)	4 (44.4)	0
Administration	32	26 (81.3)	18 (69.2)	5 (27.8)
Dietary	42	31 (73.8)	16 (51.6)	2 (12.5)
Housekeeping	19	14 (73.7)	8 (57.1)	2 (25.0)
Maintenance	14	12 (85.7)	5 (41.7)	4 (20.0)
CNA	149	115 (77.2)	78 (67.8)	20 (25.6)
LVN	66	46 (70.0)	34 (73.9)	5 (14.7)
RN	34	23 (67.6)	14 (60.9)	3 (21.4)
Other caregiver‡	49	35 (71.4)	24 (68.6)	3 (12.5)
Other/unknown‡	65	45 (69.2)	22 (48.9)	11 (50.0)
Residents	608	582 (95.7)	408 (70.1)	202 (49.5)
Age, y, mean (SD)	603	78.0 (13.3)	78.4 (13.0)	77.1 (13.0)
Sex				
F	347	332 (95.7)	237 (71.4)	121 (51.0)
M	261	250 (95.8)	171 (68.4)	81 (47.4)

*Values are no. (%) except as indicated. CNA, certified nursing assistant; COVID-19, coronavirus disease; LVN, licensed vocational nurse; RN, registered nurse. †Severe acute respiratory syndrome coronavirus 2 detected on nasopharyngeal swab tested by reverse transcription PCR. Asymptomatic infection includes confirmed COVID-19 cases with no reported typical or atypical symptoms of COVID-19. Percentage with results is equal to the number of persons with laboratory results by the number eligible in the facility. The percentage confirmed includes the number of persons with a positive PCR COVID-19 result by the number of persons with laboratory results.  ‡Other caregivers include physical therapists, respiratory therapists, rehabilitation workers, and caseworkers. Others include web developers and marketing personnel.

Varying levels of SARS-CoV-2 prevalence were identified across facilities. The lowest levels were among residents and staff in facility E (30.6% of residents [11/36], 20.0% of staff [7/35]), the highest among residents in facilities A (89.5%, 77/86), B (88.5%, 46/52), and C (87.8%, 65/74) and among staff in facilities D (26/26), F (25/25), and G (16/16) (Table 2). The prevalence of asymptomatic infection among staff members ranged from 17.4% (facility B, 4/23) to 30.6% (facility H, 11/36) (Table 2). The prevalence of asymptomatic infection among residents ranged from 19.0% (facility F, 8/42) to 85.7% (facility A, 66/77) (Table 2).

**Table 2:** Results from facilitywide testing by facility and association to the facility, Pasadena, California, USA, April 2020*

Category	Total eligible, no.	Persons tested, no. (%)	Confirmed COVID-19, no. (%)	Asymptomatic infection, no. (%)
Total	1,092	938 (85.9)	631 (67.3)	257 (40.7)
Staff	485	356 (73.6)	223 (62.6)	55 (24.7)
Residents	608	582 (95.7)	408 (70.1)	202 (49.5)
Facility A	196	174 (88.8)	123 (70.7)	79 (64.2)
Staff	109	88 (80.7)	46 (52.3)	13 (28.3)
Residents	87	86 (98.9)	77 (89.5)	66 (85.7)
Facility B	87	86 (98.9)	69 (80.2)	27 (39.1)
Staff	35	34 (97.1)	23 (67.6)	4 (17.4)
Residents	52	52 (100)	46 (88.5)	23 (50.0)
Facility C	112	109 (97.3)	90 (82.3)	34 (37.8)
Staff	35	35 (100)	25 (71.4)	6 (24.0)
Residents	77	74 (96.1)	65 (87.8)	28 (43.1)
Facility D	134	122 (91.0)	88 (72.1)	35 (39.8)
Staff	33	26 (78.8)	26 (100)	6 (23.1)
Residents	101	96 (95.0)	62 (64.6)	29 (46.8)
Facility E	98	71 (72.4)	18 (25.4)	9 (50.0)
Staff	62	35 (56.5)	7 (20.0)	2 (28.6)
Residents	36	36 (100)	11 (30.6)	7 (63.6)
Facility F	79	78 (98.7)	67 (85.9)	14 (20.9)
Staff	25	25 (100)	25 (100)	6 (24.0)
Residents	54	53 (98.1)	42 (79.2)	8 (19.0)
Facility G	117	110 (94.0)	76 (69.1)	26 (34.2)
Staff	21	16 (76.2)	16 (100)	3 (18.8)
Residents	96	94 (97.9)	60 (63.8)	23 (38.3)
Facility H	212	148 (69.8)	63 (42.6)	20 (31.7)
Staff	142	87 (61.3)	36 (41.4)	11 (30.6)
Residents	70	61 (87.1)	27 (44.3)	9 (33.3)
Facility I	57	50 (87.7)	37 (74.0)	13 (35.1)
Staff	22	20 (90.9)	19 (95.0)	4 (21.1)
Residents	35	30 (85.7)	18 (60.0)	9 (50.0)

*Results include the percentage of staff and residents who had a severe acute respiratory syndrome coronavirus 2 reverse transcription PCR test result (positive or negative). A positive result was taken as confirmation of COVID-19 infection. Asymptomatic infection was defined as a confirmed COVID-19 infection with no reported typical or atypical symptoms. Percentage of population with test results is equal to the number of persons with test results divided by the number eligible staff/residents in the facility. Percentage of confirmed COVID-19 is equal to the number of persons with a positive test result divided by the number of persons with test results. COVID-19, coronavirus disease.

## Conclusions

The ability of SARS-CoV-2 to spread rapidly among residents and staff in congregate settings poses a major infection control challenge. Our findings demonstrate a high proportion of asymptomatic infection, even within moderately affected facilities, and support the use of mass testing-based strategies in concert with symptom screening. Data from the facilitywide screenings indicate that the rate of asymptomatic infection among staff, on average, was 1 in 4, and among residents was 1 in 2.

Early in the COVID-19 pandemic, the supply of both nasopharyngeal swabs and test kits for SARS-CoV-2 rRT-PCR testing in the United States was extremely limited and made available only for symptomatic persons meeting certain criteria determined by the Centers for Disease Control and Prevention (CDC) (*12*). Diagnostic testing remained limited for many weeks, and LTCFs relied on symptom screening to exclude potentially infectious staff from work. On March 30, CDC published a change for the COVID-19 period of exposure risk from onset of symptoms to 48 hours before symptom onset (*13*). This change meant that symptom screening alone could be insufficient in protecting LTCF residents from contracting COVID-19 from asymptomatic, but infectious, staff, and studies have suggested a role for asymptomatic transmission in COVID-19 outbreaks (*14*).

Our findings demonstrate a high prevalence of both symptomatic and asymptomatic COVID-19 infection among residents and staff in 9 LTCFs. Because the potential for asymptomatic transmission of SARS-CoV-2 is concerning, for greater effectiveness, infection control efforts in LTCFs should include both mass testing–based strategies and symptom screening.
